# Advancing Clinical Information Systems: Harnessing Telemedicine, Data Science, and AI for Enhanced and More Precise Healthcare Delivery

**DOI:** 10.1055/s-0044-1800730

**Published:** 2025-04-08

**Authors:** Bernhard Pfeifer, Sabrina B. Neururer, Werner O. Hackl

**Affiliations:** 1 Division for Digital Health and Telemedicine, UMIT TIROL - Private University for Health Sciences and Health Technology, Hall in Tirol, Austria.; 2 Department of Clinical Epidemiology, Tyrolean Federal Institute for Integrated Care, Tirol Kliniken GmbH, Innsbruck, Austria.

**Keywords:** Medical informatics, International Medical Informatics Association, Yearbook, Clinical Information Systems, Artificial Intelligence, Data Science, Telemedicine

## Abstract

**Objective** : In this synopsis, the editors of the Clinical Information Systems (CIS) section of the IMIA Yearbook of Medical Informatics overview recent research and propose a selection of best papers published in 2023 in the CIS field.

**Methods** : The CIS section editors utilize a systematic approach to collect relevant articles and determine the best papers for the section. Last year, they refined the query to include the topic of telemedicine. Through a multi-stage systematic selection process, the editors reduced the initial pool to 15 candidate papers. Each of these papers underwent at least six independent reviews, culminating in a selection meeting with the IMIA Yearbook editorial board, where the three best papers for the CIS section were chosen.

**Results** : The query was carried out in January 2024 retrieving 4,784 unique papers from PubMed and Web of Science, spanning 1,401 journals. The top journals included “Telemedicine Journal and e-Health” and “Journal of Medical Internet Research”. Publications predominantly originated from the United States and United Kingdom. Significant contributions included advancements in predictive analytics, such as scalable models for diagnosis prediction and patient readmission, integration of digital twin technology, and improvements in data interoperability and security. The analysis underscores the continued focus on leveraging electronic health record data and the importance of patient-centered technologies in CIS.

**Conclusions** : These findings highlight the ongoing evolution and potential of CIS technologies in enhancing patient care, emphasizing the importance of integrating innovative solutions and patient-centered approaches in the field.

## 1. Introduction

As editors of the Clinical Information Systems (CIS) section, we annually employ a systematic approach to collect articles and select the best of them for the IMIA Yearbook of Medical Informatics. Last year, we refined the query used over the previous eight years by incorporating the topic of telemedicine and excluding terms related to geographic information systems. The inclusion of telemedicine as a search term led to a substantial increase in the number of papers found. In our synopsis of the CIS section, we highlighted the growing convergence between CIS and telemedicine, with mHealth technologies playing a significant role in healthcare delivery [ 1 ].

As in the previous years [ 2 3 4 5 6 ], the selected papers underscored the practical impact of research efforts, emphasizing patient-centric outcomes and benefits. From intelligent mobile health monitoring systems to AI-assisted decision-making in healthcare, the focus remained on improving patient care and outcomes.

Data science applications, particularly in the secondary use of clinical data, increasingly made a mark in CIS research. Papers discussing the secondary use of clinical data and the integration of machine learning and natural language processing techniques showcased the potential for innovative data-driven solutions in healthcare. The integration of artificial intelligence (AI) in healthcare informatics, especially through mHealth apps, showed promising potential for decision-making, data analysis, and personalized treatment plans. AI technologies were leveraged to support clinicians in diagnostic decision-making and enhance patient monitoring systems.

As the field of CIS continued to evolve, it became crucial to address ethical challenges surrounding AI, ensure transparency and accountability, and eliminate biases to harness its full potential in improving healthcare delivery. This highlighted the importance of ethical considerations in the implementation of AI technologies in healthcare settings.

Overall, the selected papers in 2023 reflected the ongoing advancements in technology-driven healthcare solutions, with a strong emphasis on patient-centered care, data-driven decision-making, and the integration of innovative technologies to enhance healthcare delivery and outcomes [ 1 ]. And of course, the aftershocks of the wave of publications caused by Covid-19 [ 7 ] were still clearly detectable.

We were eager to see if these developments would continue this year and if we would identify new aspects. Additionally, we were interested in examining the publication landscape concerning the special topic for the 2024 edition, “Digital Informatics for Precision in Prevention”.

## 2. About the Paper Selection

The process of searching for relevant publications and selecting the best papers in the CIS section follows a well-defined systematic approach. We reused the refined query from 2023 to search for papers [ 1 ] in mid-January 2024. We retrieved 4,784 unique papers, with 4,401 from PubMed (1,540 from the legacy query and 2,861 from the telemedicine-related search terms) and an additional 383 papers from Web of Science ^®^ (WoS). These articles were published in 1,401 journals, and Table 1 presents the 15 top-ranked journals with the highest number of resulting articles.

**Table 1.** Number of retrieved articles for Top-15 ranked journals.

Rank	Journal	Number of papers
1	Telemedicine Journal and e-Health: The official Journal of the American Telemedicine Association	268
2	Journal of Medical Internet Research	184
3	Journal of Telemedicine and Telecare	150
4	BMC Health Services Research	96
5	Digital Health	65
6	PLOS One	64
7	International Journal of Medical Informatics	62
8	International Journal of Environmental Research and Public Health	61
9	Health Communication	54
10	Frontiers in Public Health	49
11	JMIR Formative Research	46
12	Applied Clinical Informatics	45
13	Journal of the American Medical Informatics Association	41
14	Sensors (Basel, Switzerland)	40
14	BMJ Open	40

The distribution of countries of origin for each publication was similar to previous years. The United States and England again accounted for over 70% of all publications. The ranking is as follows: 1st United States (41.6%, n=1,991), 2nd England (28.9%, n=1,382), 3rd Canada (7.0%, n=333), 4th Switzerland (5.1%, n=244), 5th Germany (4.0%, n=193) and 6th The Netherlands (3.6%, n=170), Ireland (2.3%, n=110) and Australia (2.2%, n=106) round off the list of countries with more than 100 publications each.

As in previous years, we used the online systematic review tool RAYYAN 1 for the blinded, multi-stage selection process of the best papers. Each section editor screened all publications starting with an exclusion of papers considering the titles and keywords, the journals in which they were published, and finally the abstracts.

The agreement rate among all three section editors was notably high at 96.9%. We unanimously agreed to exclude 4,632 articles and include four, resulting in a Light's Kappa (for three raters) of 0.189.

Our agreement rates between pairs of editors were correspondingly higher, ranging from 97.7% (BP::WH) to 98.1% (WH::SN, Cohen's Kappa for two raters: 0.267). Four publications, which all three of us decided in favor of including in the next selection round, plus 26 publications for which two of us agreed on inclusion, as well as a further 125 publications, each of which received one vote, were screened further in the subsequent selection stages.

This process involved mutual agreement and, in several cases, full-text reviews, ultimately resulting in a shortlist of 15 publications as candidate best papers.

Twelve candidate best papers originated from the PubMed-CIS query, two were from telemedicine-related articles, and one came from the WoS query. Each candidate paper underwent a rigorous review process, with at least six independent reviews collected for each paper.

The final selection of the best papers occurred on May 3, 2024, in Paris, France, during a hybrid meeting of the IMIA Yearbook editorial board. After thorough deliberation, three papers were chosen as the best for the CIS section ( Table 2 ). Summaries of these papers are available in the appendix of this synopsis.

**Table 2.** Best paper selection of articles for the IMIA Yearbook of Medical Informatics 2024 in the “Clinical Information Systems” section. The articles are listed in alphabetical order of the first author's surname.

**Best Papers: Section Clinical Information Systems** • Mukherjee, P., Humbert-Droz, M., Chen, J. H., & Gevaert, O. (2023). SCOPE: predicting future diagnoses in office visits using electronic health records. Scientific reports, 13(1), 11005. https://doi.org/10.1038/s41598-023-38257-9 • Raab, R., Küderle, A., Zakreuskaya, A., Stern, A. D., Klucken, J., Kaissis, G., Rueckert, D., Boll, S., Eils, R., Wagener, H., & Eskofier, B. M. (2023). Federated electronic health records for the European Health Data Space. The Lancet. Digital health, 5(11), e840–e847. https://doi.org/10.1016/S2589-7500(23)00156-5 • Theodorou, B., Xiao, C., & Sun, J. (2023). Synthesize high-dimensional longitudinal electronic health records via hierarchical autoregressive language model. Nature communications, 14(1), 5305. https://doi.org/10.1038/s41467-023-41093-0

## 3. Findings and Trends: Clinical Information Systems and Telemedicine Research 2023

To manage the abundance of publications in the CIS section, we have employed additional methods such as text mining and bibliometric network visualization for several years [ 8 , 9 ]. These techniques facilitate the efficient extraction of relevant information from a large corpus of articles, allowing for a rapid understanding of their content and meaningful comparisons between them. Moreover, the visualizations depict the relationships between publications and their content, helping to identify clusters of terms and emerging trends in the field.

### 3.1. Overview of the content of all found CIS papers

**Figure 1.**
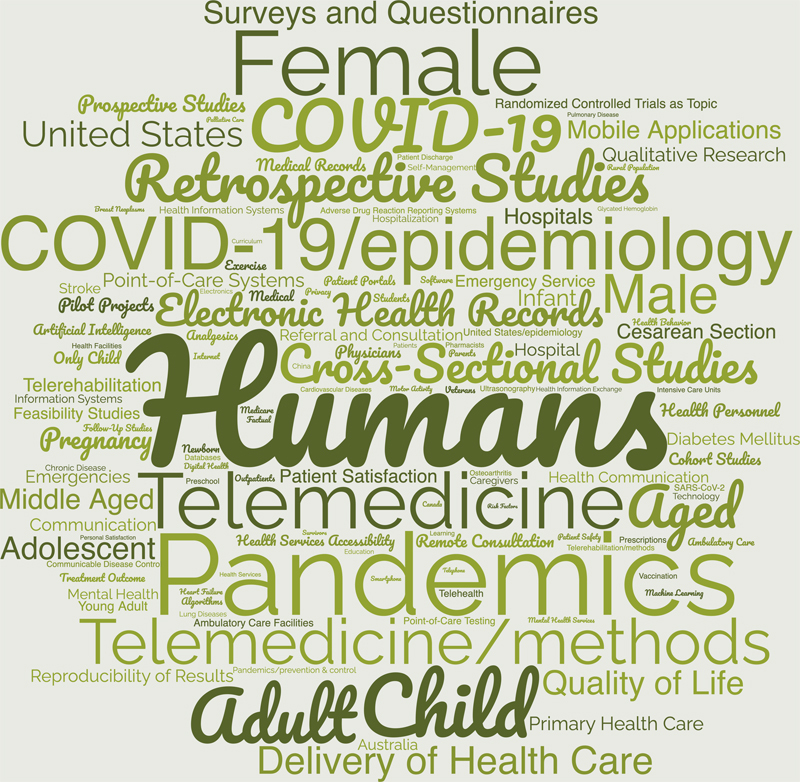
Tag cloud illustrating the frequency authors' keywords (top-250 keywords, out of n=33,516, are shown) within the 4,784 papers from the CIS query result set. Font size corresponds to frequency (the most frequent keyword was “humans” - n=3,353).

In previous years, the creation of a tag cloud from the authors' keywords to get an initial overview of the content of the individual publications has proven its worth. This year, we could extract 33,516 keywords from all articles and created the tag cloud with the top-250 of them ( Figure 1 ). It is almost identical to last year [ 1 ].

**Figure 2.**
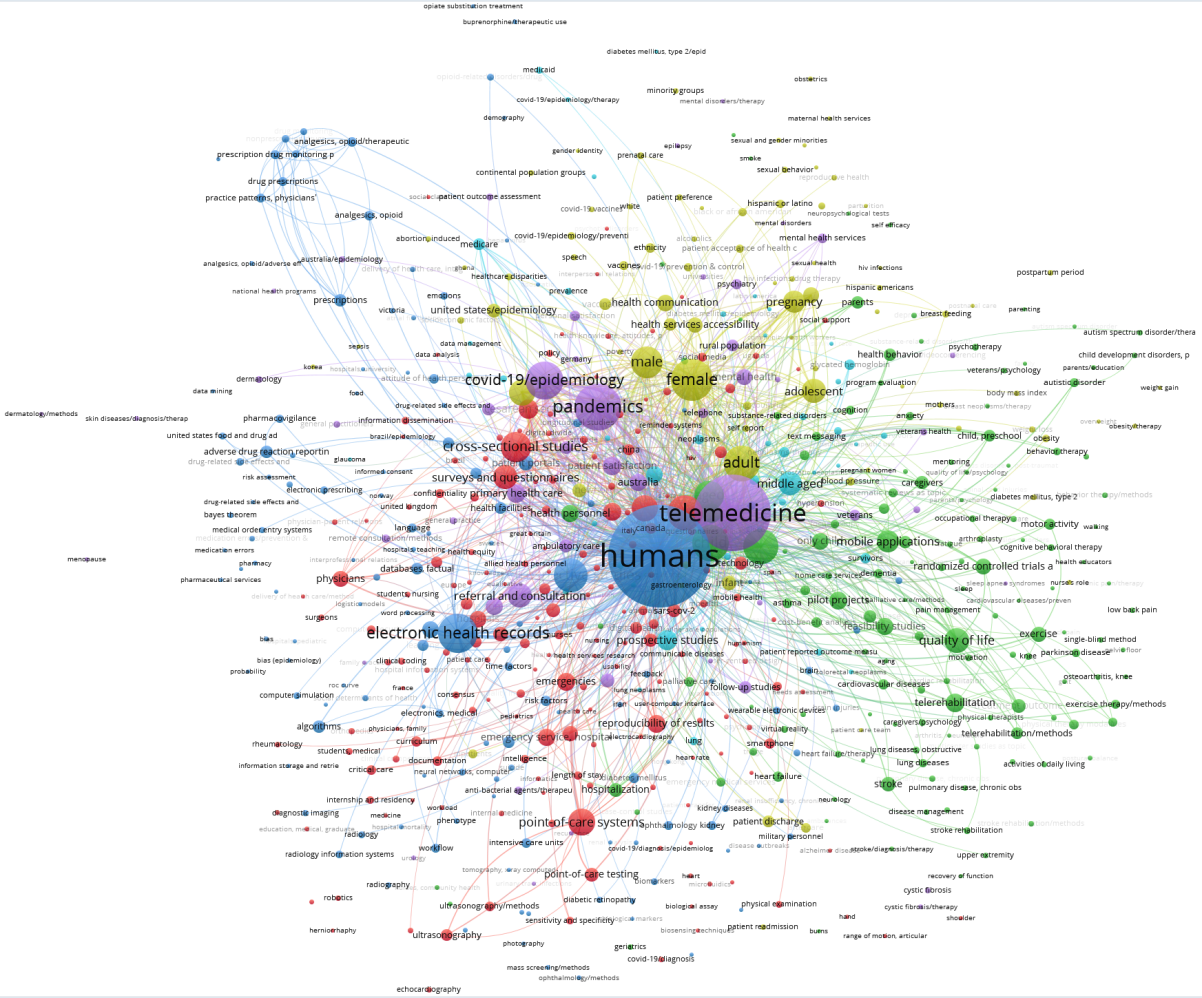
Clustered co-occurrence map of the top-644 keywords (keywords with the greatest total link strength, n=644 of 5,184 distinct keywords) from the 4,784 papers in the 2024 CIS query result set. Only keywords that we found in at least six different papers were included in the analysis. Node size corresponds to the frequency of the keywords (“humans”: n=3,914). Edges indicate co-occurrence (only the top 1,000 of 19,454 edges are shown). The distance between nodes corresponds to the link strength between the keywords. Colors represent the six different clusters. The network was created with VOSviewer [ 8 ].

Analog to last year's approach, we also created a bibliometric network in addition to the tag cloud to be able to show details of connections and interrelations between the publications. We again used the tried and tested tool VOSviewer [ 8 ] with the same settings as last year to create a clustered co-occurrence map of the keywords, which is depicted in Figure 2 .

**Figure 3.**
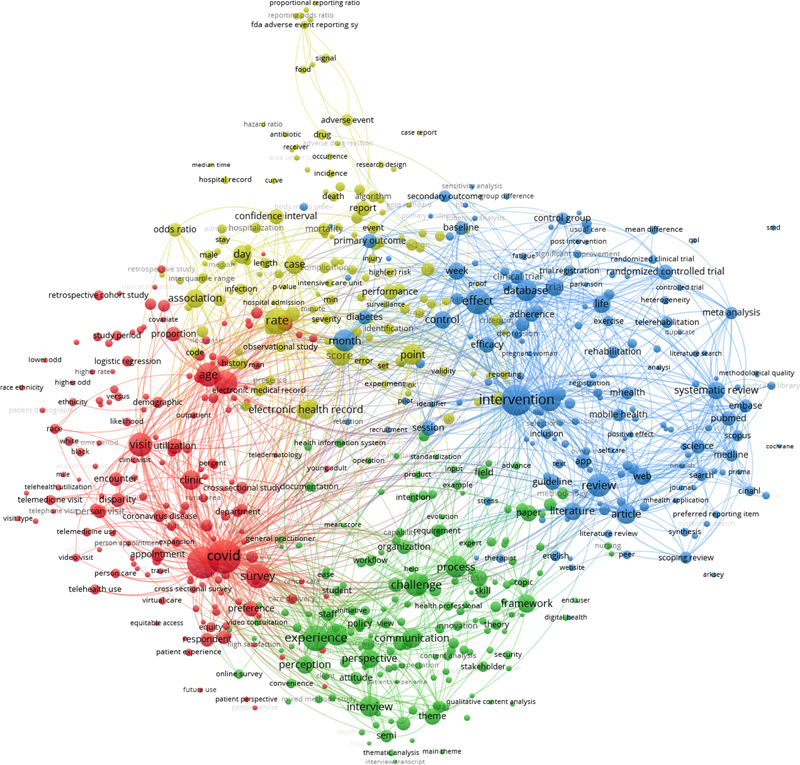
Clustered co-occurrence map of the top-653 terms (top-60% of the most relevant terms: n=653 of 92,185) from the titles and abstracts of the 4,874 papers in the 2024 CIS query result set. Only terms that we found in at least 25 different papers were included in the analysis. Node size corresponds to the frequency of the terms (binary count, once per paper, “covid”: n=1,242). Edges indicate co-occurrence (only the top 1,000 of 125,135 edges are shown). The distance between nodes corresponds to the association strength of the terms within the texts. Colors represent the four different clusters. The network was created with VOSviewer [ 8 ].

The resulting picture is very similar to last year's, with six different clusters containing the different perspectives and aspects of the studies. To further ensure consistency with previous years' investigations, a parallel analysis was conducted using the terms extracted from all publications' titles and abstracts. The resulting co-occurrence map of the top-653 terms is visualized in Figure 3 .

The overall picture remains largely unchanged from last year, with four clusters exhibiting similar elements and structures. Consequently, little new information can be gleaned from this high-level analysis. Therefore, a detailed examination of the central publications in our CIS selection is necessary and we present a detailed analysis of the candidate papers and best papers next.

### 3.2. Insights into the Candidate Papers and Best Papers

The following summaries highlight the significant contributions and present the insights that we gained from our analysis of the actual scientific landscape in the field of CIS.

Secondary use of health and real-world data continues to be the central field of research and activity around CIS and several of the candidate papers can be assigned to this area. So, the first of the best papers in the 2024 CIS selection, a contribution by Mukherjee et al., which explores the development of SCOPE, an interpretable and scalable machine learning model designed to predict likely diagnoses during office visits using electronic health records (EHRs) and addresses the critical challenge of leveraging EHR data to predict patient diagnoses efficiently and accurately, offering a practical solution that balances performance with interpretability. This is crucial for enhancing clinical decision support systems and reducing physician burnout associated with EHR documentation [ 10 ].

Predictive analytics is also the subject of a candidate paper, the contribution by Khodadadi et al., who introduce Patient Forest, a novel machine learning model leveraging deep random forest techniques to predict patient readmission and mortality using EHRs. By employing a cascade structure of random forests, Patient Forest effectively learns patient representations from high-dimensional EHR data, outperforming traditional machine learning models like Support Vector Machines, Logistic Regression, and standard Random Forests, particularly when data is limited. The model demonstrated superior performance on two extensive datasets, MIMIC-III and eICU, showing its robustness and applicability across different healthcare settings. The study's significance lies in its potential to enhance predictive analytics in CIS, leading to improved patient management and outcomes by providing a scalable and interpretable tool for clinicians to anticipate critical events such as readmissions and mortality. This approach addresses key challenges in EHR data utilization, including handling missing data and integrating diverse data types, thereby advancing the field of clinical decision support systems [ 11 ].

Fishing in the same waters, clinical prediction tasks such as mortality prediction and length of stay forecasting, are Basnet et al. who introduce RIMD, a novel deep learning model designed to handle the challenges of sparse, noisy, and imbalanced EHRs for such prediction tasks. The RIMD model incorporates a decay mechanism, modular recurrent networks, and a custom loss function to effectively learn from patterns in sparse data, select relevant inputs dynamically, and manage class imbalance. Using the MIMIC-III dataset, RIMD demonstrated superior performance in F1-Score, AUROC, and PRAUC compared to existing models. The significance of this study lies in its potential to improve predictive accuracy and generalization on non-independent and identically distributed data, addressing critical issues in clinical data analysis and enhancing the utility of CIS for better patient management and outcomes [ 12 ].

Boussina et al. are advancing the field of prediction one step further toward real-time prediction in healthcare settings. They present the development and deployment of a scalable, cloud-based healthcare predictive analytics platform capable of integrating seamlessly into existing EHR systems. This platform, deployed at UC San Diego Health, extracts and processes EHR data in real-time and feeds back actionable insights into the EHRs to support clinical decision-making. A key demonstration of its capabilities includes the implementation of a deep learning model for early sepsis prediction, which showed promising performance during a 6-month silent-mode evaluation. The platform's architecture emphasizes security, interoperability, and high availability, leveraging Amazon Web Services infrastructure to ensure robustness and scalability. The deployment of this platform addresses significant challenges in translating predictive analytics into clinical practice by providing a real-time, reliable, and integrated solution [ 13 ].

A very interesting work from a methodological perspective comes from Li et al. Their article presents Hi-BEHRT, a novel deep learning model designed to improve the prediction of clinical events using extensive and complex EHRs. Hi-BEHRT leverages a hierarchical transformer architecture to process long sequences of multimodal data, such as diagnoses, medications, procedures, tests, and lifestyle factors, over a patient's medical history. This model significantly expands the receptive field of traditional transformers, enabling it to capture long-term dependencies in the data and thereby improve predictive accuracy for diseases like heart failure, diabetes, chronic kidney disease, and stroke [ 14 ].

The development and testing of new AI methods requires the best possible, quality-assured, and freely available data sets. However, these are not always easy to find due to various restrictions, especially in data protection. The second of the best papers in the 2024 CIS selection deals with this topic. Theodorou et al. introduce the Hierarchical Autoregressive Language mOdel (HALO) for generating high-dimensional, longitudinal synthetic EHRs. HALO captures the complex patterns and relationships in EHR data, preserving statistical properties and enabling the training of accurate machine learning models without privacy concerns. HALO generates realistic patient records by modeling disease code probabilities, clinical visits, and patient histories, achieving high fidelity with a correlation coefficient above 0.9R ^2^ compared to real EHR data. Extensive experiments demonstrate HALO's superiority in generating high-fidelity data, enhancing predictive modeling accuracy, and supporting machine learning models with synthetic data that mirror real EHR performance. This model addresses significant challenges in EHR data generation, including handling high-dimensionality and preserving patient privacy, making it a valuable tool for advancing clinical predictive modeling and health informatics research [ 15 ].

Vallée shows another aspect in this area and highlights the transformative potential of digital twin technology in healthcare. By integrating real-time data, advanced analytics, and virtual simulations, digital twins offer significant improvements in patient care, predictive analytics, clinical operations, and training. This technology enables the collection and analysis of extensive patient data, allowing for personalized treatment plans and proactive health management. The relevance of this publication for CIS lies in its detailed exploration of how digital twins can optimize clinical workflows, enhance patient safety, and drive innovation, thereby improving overall healthcare outcomes and efficiency [ 16 ].

Addressing data integration and interoperability towards FAIR (Findable, Accessible, Interoperable, Reusable) data is a significant focus in the field of CIS. Many publications in our result set deal extensively with these aspects, which is also reflected in the candidate papers. Sinaci et al. for example present a methodology and tool for transforming existing health data into HL7 FHIR format while ensuring compliance with FAIR principles. This approach facilitates data sharing and integration across different healthcare and research institutions by addressing interoperability challenges. The study demonstrated that the methodology could effectively convert health data into a FAIR-compliant format, enhancing data utility and accessibility for research and clinical use [ 17 ].

Various ideas and concepts for health data spaces showing the need for balancing data accessibility with privacy and security were also found among the CIS publications. In particular, the third of the best papers, a contribution by Raab et al., discusses the European Commission's draft for the European Health Data Space (EHDS), which aims to empower citizens to access and share their personal health data with healthcare providers while ensuring the secondary use of this data for research and development. The authors propose a federated personal health data space architecture that stores data on personal devices rather than centralized silos, enhancing privacy and control for citizens. This approach aligns with the European General Data Protection Regulation and encourages transparency and trust, thereby promoting active participation in data sharing and research [ 18 ].

However, this will also require applicable standards in practice. Pedrera-Jiménez et al. explore the question of whether OpenEHR, ISO 13606, and HL7 FHIR can work together. They conclude that while each standard has unique strengths and weaknesses, they can indeed work together in a complementary fashion. OpenEHR is best suited for comprehensive data modeling and persistence, ISO 13606 excels in complex data exchange and semantic interoperability, and HL7 FHIR is optimal for agile, point-to-point data exchange and implementation resources [ 19 ].

Meredith et al. present an architectural approach using the FOXS stack — comprising HL7 FHIR, OpenEHR, IHE XDS, and SNOMED CT — to support next-generation digital care records that align with FAIR principles. The study demonstrates that the FOXS components broadly conform to the European Open Science Cloud Interoperability Framework for semantic interoperability, ensuring that health data can be effectively used for both clinical and research purposes [ 20 ].

Patient-centeredness is an ever-increasing development in the CIS sector and wearables have long since found their way into the field of CIS. They provide important, so to say patient-generated data for collaborative health monitoring and decision support in healthcare. However, the integration of this data also brings with it many challenges. Pathak et al. present a plug-and-play device, called SemBox, designed to enable semantic interoperability among heterogeneous wearable health monitoring devices. SemBox connects wirelessly to various health monitoring devices, receives data packets, and uses a Mamdani-based fuzzy inference system for data classification [ 21 ].

The integration of patient-reported outcomes and patient-generated data with clinical data to provide comprehensive support is the goal of the EU-funded CAPABLE project. Lanzola et al. explore a multi-agent system, designed within this project, to provide coaching advice to cancer patients and clinical decision support to their healthcare providers. The system utilizes HL7 FHIR to ensure semantic interoperability among agents, coordinating their activities through a Case Manager that monitors a shared data platform for triggering conditions. The Case Manager dynamically notifies agents when relevant data patterns are detected, thus orchestrating the agents' actions based on updated patient information. The approach was validated through clinical scenarios, confirming the Case Manager's effectiveness in managing agent interactions [ 22 ].

Patient portals allow patients to access their health information and medical records online, thereby promoting patient engagement and enabling patient-centered care. Kuppanda and Jenkins investigate the experiences and attitudes of patients in the UK towards patient portals. The qualitative, grounded theory study found that patients generally have positive perceptions of using patient portals, citing benefits such as improved access to information, convenience, and enhanced communication with healthcare providers. However, the study also identified several areas needing improvement, such as better feature awareness among patients, more consistent portal services across different providers, and increased support for users with low technological literacy. These findings are highly relevant for the development and implementation of CIS as they highlight the importance of user-friendly design, comprehensive patient education, and the need for uniform service standards to ensure the successful adoption and utilization of patient portals [ 23 ].

The last paper among the 2024 CIS candidates deals with the topic of data and system security in a very interesting way. He et al. explore a novel AI-based ethical hacking method designed to enhance the security of health information systems. By implementing an optimized ethical hacking framework using the ant colony optimization algorithm, the study compares the performance of AI-based and traditional ethical hacking methods in a simulated health information system environment using OpenEMR. The optimized method significantly outperformed the unoptimized method in terms of time efficiency, success rate of exploits, and number of successful attack paths identified. Key vulnerabilities discovered included remote code execution, cross-site request forgery, and improper authentication, among others. This approach demonstrates the potential of AI in systematically identifying and addressing security weaknesses in CIS, thus enhancing the overall cybersecurity posture of healthcare organizations [ 24 ].

Of course, we also considered the CIS publications set given the special topic for the 2024 edition, “Digital Informatics for Precision in Prevention”, and found some interesting contributions, for example a “Clinician's Guide to Running Custom Machine-Learning Models in an Electronic Health Record Environment” by Ryu et al. [ 25 ], and a systematic methodological review on prediction models using AI and longitudinal data from EHRs by Carrasco-Ribelles et al. [ 26 ].

As in previous years, we would like to draw attention to this year's survey paper from the CIS section. Peek et al. have reviewed recent literature on the clinical deployment of AI-based clinical decision support systems [ 27 REF. Peek N, Capurro D, Rozova V, and van der Veer SN. Bridging the Gap: Challenges and Strategies for the Implementation of Artificial Intelligence-based Clinical Decision Support Systems in Clinical Practice - Yearbook 2024. doi: 10.1055/s-0044-1800727].

## 4. Conclusions and Outlook

In conclusion, the 2024 synopsis of the CIS section of the IMIA Yearbook of Medical Informatics highlights significant advancements and ongoing challenges in leveraging EHR and real-world data for improved patient care. From predictive analytics to the development of scalable, real-time platforms, the selected papers showcase innovative approaches to enhancing clinical decision support systems. Notable contributions include interpretable machine learning models for diagnosis prediction, novel techniques for patient readmission and mortality forecasting, and advanced methods for handling sparse and imbalanced EHR data. The integration of digital twin technology, FAIR data principles, and methodologies for data interoperability further emphasizes the transformative potential of CIS.

Moreover, the focus on patient-centered solutions, such as wearable health monitoring devices, patient portals, and multi-agent systems for personalized care, underscores the importance of involving patients in their healthcare journeys. The exploration of ethical hacking techniques to improve system security demonstrates the field's commitment to safeguarding sensitive health information.

This comprehensive review, combined with the recommended survey paper by Niels Peek and colleagues, provides valuable insights into the current landscape and future directions of CIS. As we continue to navigate the complexities of AI integration in healthcare, these studies offer practical solutions and strategic guidance for advancing clinical practice and improving patient outcomes. We highly encourage readers to delve into these findings and consider their implications for the ongoing development and deployment of CIS technologies.

## Footnotes

1
https://www.rayyan.ai
